# The Effect of Tensile Stress on the Conformational Free Energy Landscape of Disulfide Bonds

**DOI:** 10.1371/journal.pone.0108812

**Published:** 2014-10-06

**Authors:** Padmesh Anjukandi, Przemyslaw Dopieralski, Jordi Ribas–Arino, Dominik Marx

**Affiliations:** 1 Lehrstuhl für Theoretische Chemie, Ruhr–Universität Bochum, Bochum, Germany; 2 Faculty of Chemistry, University of Wroclaw, Wroclaw, Poland; University of Leeds, United Kingdom

## Abstract

Disulfide bridges are no longer considered to merely stabilize protein structure, but are increasingly recognized to play a functional role in many regulatory biomolecular processes. Recent studies have uncovered that the redox activity of native disulfides depends on their C–C–S–S dihedrals, 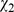 and 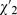. Moreover, the interplay of chemical reactivity and mechanical stress of disulfide switches has been recently elucidated using force–clamp spectroscopy and computer simulation. The 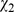 and 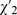 angles have been found to change from conformations that are open to nucleophilic attack to sterically hindered, so–called closed states upon exerting tensile stress. In view of the growing evidence of the importance of C–C–S–S dihedrals in tuning the reactivity of disulfides, here we present a systematic study of the conformational diversity of disulfides as a function of tensile stress. With the help of force-clamp metadynamics simulations, we show that tensile stress brings about a large stabilization of the closed conformers, thereby giving rise to drastic changes in the conformational free energy landscape of disulfides. Statistical analysis shows that native TDi, DO and interchain Ig protein disulfides prefer open conformations, whereas the intrachain disulfide bridges in Ig proteins favor closed conformations. Correlating mechanical stress with the distance between the two 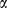
–carbons of the disulfide moiety reveals that the strain of intrachain Ig protein disulfides corresponds to a mechanical activation of about 100 pN. Such mechanical activation leads to a severalfold increase of the rate of the elementary redox 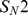 reaction step. All these findings constitute a step forward towards achieving a full understanding of functional disulfides.

## Introduction

Disulfide bonds are known since long to play crucial roles in the workings of Nature's protein machinery [1]. For many years it has been accepted that disulfide bonds have been added during evolution to enhance the stability of proteins that function in a vividly fluctuating cellular environment by establishing covalent crosslinks [2], thus protein folding and disulfide bond formation going hand in hand [3]. However, recent evidence indicates that disulfide bonds can be more than just inert structural motifs [4]. Today, the emerging paradigm is that the disulfide proteome consists of two subproteomes, a “structural” and a redox–sensitive, “functional” class as many disulfide bonds have been discovered to play crucial roles in regulating the biological activity of the proteins themselves [5]–[8]. Such so–called “disulfide switches” are enabled by the very chemistry of sulfur bonds, which can easily undergo redox reactions at mild, physiological conditions [9]–[12]. Moreover, since a few years only, we have witnessed a growing interest in probing experimentally the cleavage and interchange reactions of functional disulfides under controlled stress conditions in order to fully understand the molecular basis of these elementary biological processes [13]–[16].

**Figure 1:**
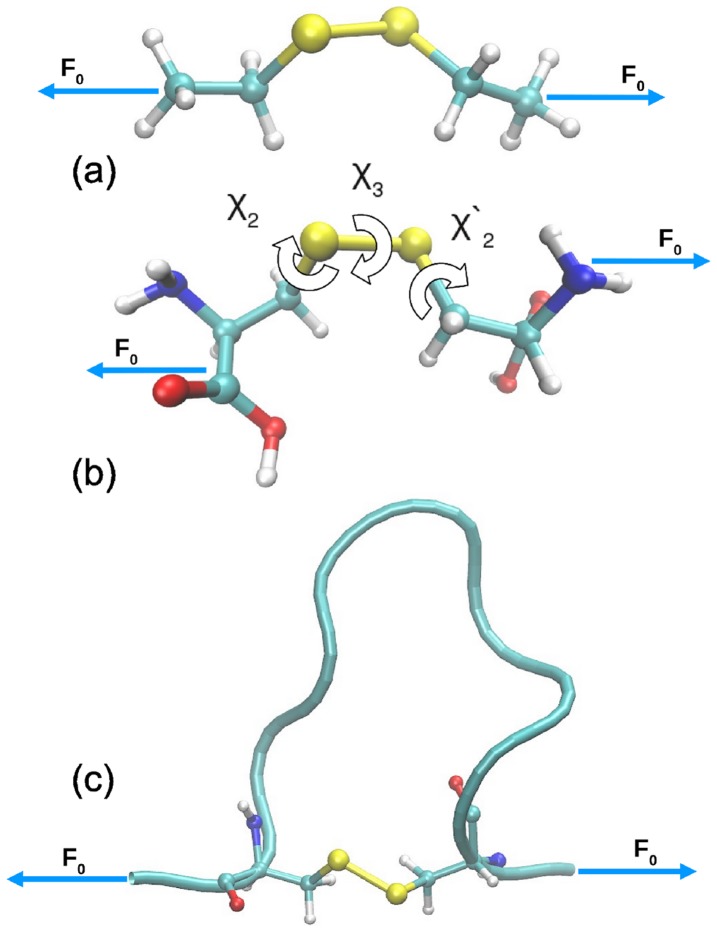
Three models of increasing complexity used to investigate strained disulfide bonds. (a) Diethyl disulfide (DEDS), (b) cystine, and the (c) polypeptide model (see text). The collinear constant force of magnitude 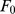 is applied to the terminal methyl C atoms in panel a and to N and C termini in panels b and c. The dihedral angles 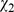, 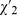 and 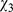 are defined in panel b.

Most interestingly, it has been observed that numerous “disulfide switches” are associated with strained conformations involving the C–C–S–S dihedral angles, i.e. 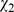 and 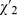 as defined in Fig. 1, in response to geometrical constraints imposed by proteins in certain regions of their structures, such as e.g. in the case of staple conformations that store a significant amount of torsional strain energy [8]. Since the value adopted by 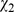 and 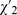 is seemingly a factor that controls the ability of a given disulfide to act as a switch, the energy landscape of disulfide bridges for hindered torsional motion around their 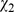 and 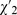 angles has recently raised much interest [17], [18]. In particular, the potential energy surface (PES) for the torsion around these dihedral angles has been computationally investigated in a small model system (diethyl disulfide, DEDS) to scrutinize the conformational energetics of the disulfide bridge [17], [18]. Upon mapping the distribution of the disulfide bonds in the Protein Data Bank (PDB) onto the computed two-dimensional PES, an astonishing conformational diversity of disulfide bridges in terms of their preferred 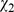 and 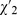 values has been observed [17], [18]. Although the majority of disulfides adopt the lowest energy conformation, a significant amount of disulfides is found in other regions of this PES (including other local minima and non-stationary points) [17], [18]. This computational and statistical analysis on the conformational landscape of disulfide bridges has certainly delivered useful information as to know which disulfides are likely to be redox–active. However, recent work on the mechanochemistry of disulfide bridges suggests that another variable should be explicitly included in this analysis in order to have a more complete picture of the interplay between conformation and redox activity of disulfides.

Research on the mechanochemistry [19]–[21] of disulfide bridges [22] has already provided spectacular insights into the intimate coupling of chemical reactivity and mechanical stress [23], [24]. Along such lines, recent pioneering force–clamp atomic force microscopy (AFM) experiments on a mutated I27 domain of cardiac titin have revealed an abrupt switch in the redox reactivity of disulfide bonds when stretching the peptide with external forces of roughly 0.5 nN [25]. Enormous efforts have been invested to unravel the molecular origin of this most enigmatic switch and to decipher the mechanical properties of disulfides in general [25]–[34]. Using again a minimal molecular model system, namely DEDS in bulk water, it has been demonstrated that it is not the stretching–induced distortions of the central C–S–S–C dihedral angle (denoted as 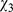 in the protein literature), but stress–induced conformational change of the C–C–S–S dihedrals (i.e. 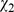 and 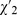 in Fig. 1) which regulates the measured redox activity [34]. In the absence of force, the disulfide bond is in a conformational state that is favorable for nucleophilic attack in the sense of a standard 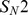
–type reaction and thus for redox reactions. The molecular reason is that the 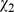 and/or 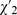 dihedral in the unstretched molecule is in the so-called “open” state and allows for ready collinear attack of the nucleophile as explained earlier [34]. However, upon straining the molecule by stretching it, both angles are quickly deformed towards 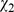/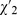 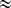 180°/180°, corresponding to the so-called “closed/closed” state, which blocks the reactivity cone for 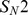 attack and thus counteracts the redox activity. This change from open to closed/closed conformations occurs at around 0.5 nN and provides the molecular underpinnings of the disulfide switch in the single–molecule limit [25], [34]. We mention in passing that a closed state with respect to the 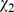 dihedrals according to this “mechanochemistry nomenclature” corresponds to a trans conformer within the conventional classification scheme. The open states, in turn, embrace the subclasses of spirals, hooks or staples, the latter category including examples of “allosteric disulfide bonds” [6], [35].

These findings suggest a possible link to the aforementioned functional disulfide bonds in native proteins which are strained in terms of the 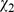/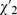 dihedrals, at the level of the underlying 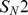
–type redox reaction. It is noted in passing that, of course, global conformational blocking and lack of solvent accessibility in the case of buried disulfides, which might hinder the approach of large nucleophiles already far away from the redox center itself, are additional important factors that certainly affect the apparent reactivity of disulfides. Having this in mind, it should be recalled that IgG4 [36], [37] and IgG2 [38] systems exhibit functional “structural isoforms” which are proposed to be mediated via intrachain disulfide bonds. Thus, apart from the solvent accessibility, the proximity of internal thiols in proteins could also mediate disulfide bond reduction, making these intrachain disulfides also interesting candidates for the reduction process.

In view of these observations obtained at the single–molecule level, it is clear that the explicit consideration of the tensile stress is mandatory for a full understanding of both the conformational landscape of disulfide bonds and its connection with their redox activity. Here, on the basis of extensive isotensional metadynamics simulations, we will evaluate how the conformational free energy surface (FES) of disulfide bridges as a function of torsions around the 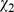 and 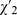 dihedral angles is transformed upon applying tensile stress. At this point, it should be emphasized that our systematic study of the conformation energetics of disulfide bridges not only incorporates the effects of external forces, but it also incorporates finite temperature and entropy effects through mapping the associated free energy surfaces.

Our analysis will reveal that tensile stress brings about drastic topological changes on the conformational FES of disulfides. Indeed, it will be demonstrated that mechanical stress results in a notable stabilization of the closed conformation of disulfide moieties and that this is the single minimum observed in the free energy surfaces obtained at forces around 1 nN. Besides, we will present the results of a new statistical analysis of Protein Data Bank (PDB) structures, which will disclose that the disulfide bonds of different classes of proteins feature markedly distinct patterns when it comes to their preferred 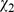 and 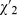 dihedrals and thus conformations. Specifically, intrachain immunoglobulin (Ig) protein disulfides strongly prefer 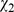/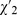 combinations around 180°/180°, i.e. closed/closed conformations, whereas functional disulfides residing in thiol–disulfide interchange proteins (TDi) [39], disulfide oxidoreductases (DO) [40] and in interchain Ig proteins most clearly avoid these values of 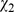 and 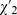. The latter classes are thus “conformationally active” disulfides, that is, disulfides that possess a local configurational arrangement close to the disulfide bridge being the redox center, that favor an easy approach of nucleophiles. It will be shown that the 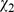/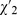 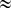 180°/180° conformations of disulfides in intrachain Ig proteins, which originate in interstrand constraints imposed by *native* Ig proteins, correspond to a tensile force on the order of 100 pN on these strained disulfides. Based on this force, which is well within the range of physiological forces relevant to biological processes [41]–[46], it can be argued that these particular disulfides are “mechanically activated”.

## Results

### From molecular models to polypeptides

At the core of our analyses will be to understand the conformational behavior of disulfide bonds depending on strain, which is imposed by external tensile stress generated by stretching the protein isotensionally, i.e. at constant force. The mutated I27 domain of cardiac titin used earlier in AFM experiments [25] is simplified to the polypeptide fragment Ile–Cys–Leu–Ser–Glu–Pro–Asp–Val–His–Cys–Gln, where the disulfide bridge connects the 2nd to the 10th cysteine residue thus forming a loop as schematically shown in Fig. 1c. This model inherits the topological essence of the I27 domain of titin [25], [26], [47] while being stretched. Furthermore, the system complexity will be reduced systematically towards the minimal system DEDS, see Fig. 1a, by also investigating a dipeptide, cystine as depicted in Fig. 1b, which will enable us to systematically refer to earlier findings [34] in the current framework. All three models are fully solvated in water at ambient conditions and stressed by applying a constant force (of magnitude 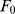 to the terminal heavy atoms as indicated in Fig. 1) using isotensional molecular dynamics and metadynamics simulations [21]. Full information on models, methods, and references is provided in File S1.

### Conformational diversity and mechanical stress

**Figure 2:**
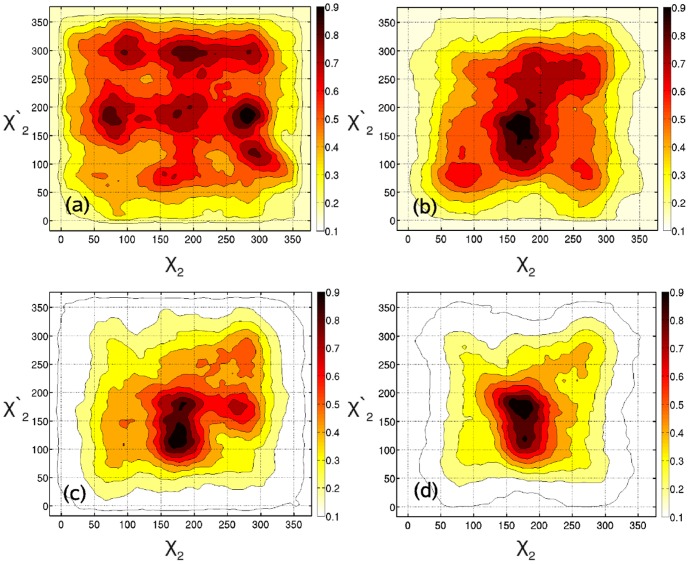
Conformational diversity of the polypeptide model depending on tensile force. Probability distribution functions 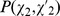 obtained from Boltzmann–inversion of the two–dimensional free energy landscapes from metadynamics simulations for the polypeptide model at zero force and 0.3, 0.6, and 1.0 nN in panels a to d, respectively.

What happens to the conformational degrees of freedom when putting disulfide bonds under tensile stress, i.e. upon mechanical stretching our polypeptide model? As clearly displayed in panel a of Fig. 2, the sampled probability distribution function associated with the 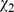 and 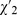 dihedral angles (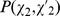) features a pronounced conformational diversity of the *unstretched* molecule. In particular, with an overwhelming probability at least one of the two C–C–S–S dihedrals is in a conformation that is open to undergo redox reactions. The picture changes significantly already at a stretching force of 0.3 nN in panel b, with a strong preference of 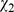/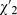 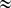 180°/180° such that closed/closed conformations are found to be significantly populated. This trend becomes systematically more pronounced upon stressing the molecule up to 1 nN (cf. panel d), where essentially exclusively the highly strained closed/closed state is seen to survive. The same observation is made for the two smaller models, DEDS and cystine, where closed/closed conformations are overwhelmingly populated at a force of 300 pN and higher, see Figs. S3 and S4 in file S1. Thus, we infer that the external force definitely impacts on the conformational preference of the 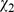 and 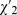 dihedrals in disulfide systems in the sense of greatly reducing their conformational diversity towards the closed/closed state. In addition, the two smaller systems support the key characteristics found for the polypeptide and are thus valuable reduced models for subsequent analyses. The force-induced stabilization of the closed/closed conformation can be easily understood in view of the fact that this conformation leads to a more extended molecule. This, in turn, is in line with previous single–molecule force spectroscopy studies, where it was found that the elasticity of biopolymers in the low-force regime stems from changes in the values of the dihedrals of the backbone [48]–[50]. Such elastic response can in general be well captured with simple analytical models, such as the worm–like chain (WLC) or the freely–jointed chain (FJC) models [49], [50].

### Conformational diversity and mechanical coordinate

Next, the impact of stretching on the total population of open conformers is analyzed systematically in Fig. S6 in file S1, which exhibits a significant decay upon stretching, again found for all systems considered. This suggests the question if there is a simple way to connect this observation, i.e. the conformational response of the 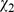 and 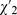 torsional degrees of freedom of the disulfide moieties to tensile force, to a structural parameter? Indeed, the so–called mechanical coordinate 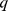, i.e. the distance between the atoms to which the external force is applied, increases qualitatively in a similar manner according to Fig. S7 in file S1. In addition, this behavior of 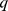 *versus* 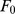 perfectly correlates with the decreasing contributions of open conformers in Fig. S6 in file S1. However, its change in absolute terms is strongly system dependent: 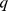 increases by about 6 Å from zero force to 2 nN in case of the polypeptide, whereas this amounts to only 3 and 2 Å for cystine and DEDS, respectively.

### Comparison to native redox disulfides

**Figure 3:**
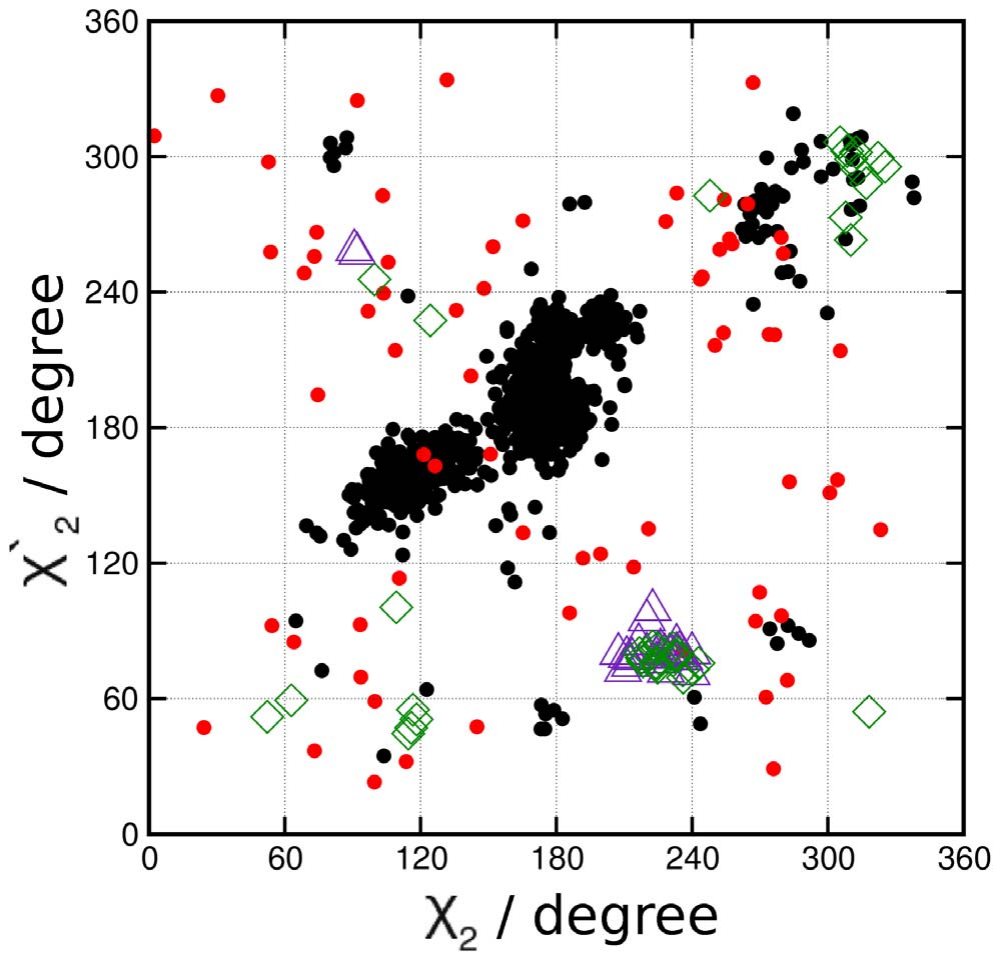
Statistical analysis of the conformational diversity of native protein disulfides. Scatter plot of the 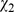/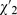 correlation for TDi (open green diamonds), DO (open indigo triangles), interchain Ig (small red circles) protein disulfides and intrachain Ig (small black circles) disulfides based on 40, 27, 69 and 927 X–ray crystal structures, respectively, obtained from the data reported in Ref. [59] that are based on analyzing the Protein Data Bank.

After having demonstrated that the conformational diversity (in particular, the open character of disulfide bonds that facilitates redox reactions via nucleophilic attack) is dramatically reduced if polypeptides as well as smaller molecules get stretched with forces of sub–nN magnitude, we will now inspect whether the native disulfides exhibit any conformational preference depending on their particular protein environment. The scatter plot of Fig. 3 reveals that TDi, DO and interchain Ig protein disulfides prefer to stay in open conformations, thus being prone towards redox processes [34]. This is in consonance with the experimental observation that these classes of proteins contain functional disulfides that readily undergo oxidation/reduction themselves and also facilitate the reduction of other disulfides, thus regulating protein function [51]. In stark contrast with the conformational trends uncovered for TDi, DO and interchain Ig protein disulfides, our statistical analysis (see Fig. 3 and Fig. S2b in file S1) shows that intrachain disulfide bonds in Ig proteins mostly prefer 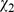/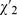 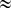 180°/180° and thus the closed/closed state [52], [53], thereby hindering redox reactions at the level of the underlying 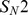
–type chemical reaction. The analysis presented above on the influence of mechanical stress on the conformational diversity of disulfides (cf. Fig. 2) suggests that this specific conformational preference can be traced back to these intrachain disulfide bridges being subjected to tensile stress. In the next subsection, we will estimate the value of tensile stress that is compatible with the systematic strain observed in this class of disulfides.

### Molecular understanding

**Figure 4:**
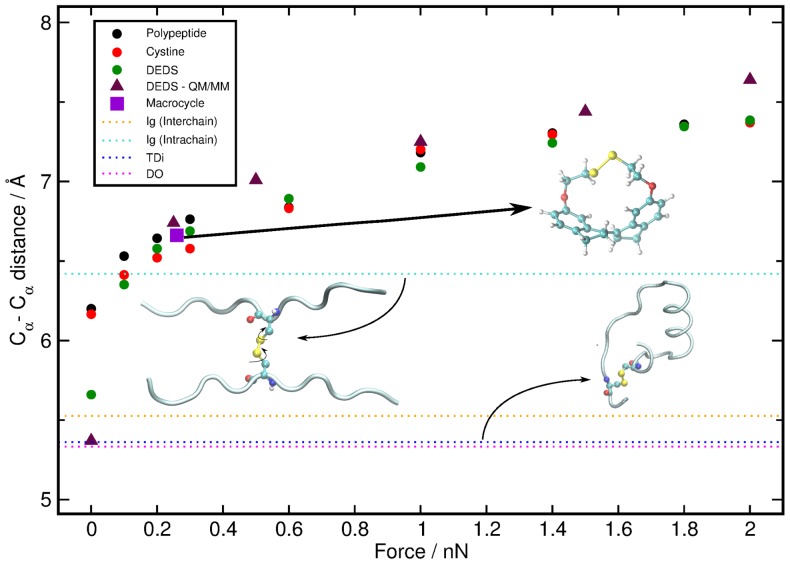
Stress–strain relation of disulfide bonds parameterized by the response of the C_α_–C_α_ distance to tensile force. Dependence of the computed average distance between the C_α_–atoms as a function of 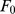 for the polypeptide model (black circles), cystine (red circles), and DEDS (green circles) depicted in Fig. 1 and obtained from force field equilibrium (at zero force) and force clamp MD (for 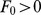 nN) simulations. Computational reference data for DEDS obtained from QM/MM simulations are shown by brown triangles and the experimental reference based on the strained macrocycle [27] (see text) is marked by a violet square. The horizontal blue, pink and orange dotted lines are the average C_α_–C_α_ distances of disulfide bonds in TDi, DO and interchain Ig proteins, respectively, whereas the cyan dotted line corresponds to intrachain Ig proteins; these averages have been computed using the identical data sets as those that underly Fig. 3, see caption.

Upon analyzing a variety of structural properties in addition to 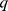, we found that the computed distance between the two 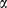
–carbon atoms of the disulfide moiety (the C
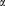
–C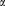 distance) correlates uniquely and quantitatively with tensile force for *all systems* investigated as revealed by the circles in Fig. 4; note that what we call “
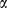
–carbon” following the usual protein nomenclature would be the “
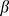
–carbon” with respect to a chemical reaction involving the disulfide bond. This correlation, first of all, suggests that the C_α_–C_α_ distance *versus* force curve can be used as a (nonlinear) “ruler” to translate tensile stress to strain within disulfide bonds, which is simply measured (or parameterized) by the separation between the C_α_–atoms. In order to gauge this correlation, we took advantage of an experimentally reported strained disulfide bond embedded in a macrocycle (see supporting Sec. I.C and Fig. S1 in File S1 for details) for which the strain–inducing tensile force is known *independently* [27] from our present calculations. For the same molecule we computed the average C_α_–C_α_ distance (square), which nicely falls on our correlation curve together with the force taken from the literature [27] without any adjustment. A further validation is obtained by computing the correlation curve for DEDS using the superior QM/MM [54] approach instead of biomolecular force field molecular dynamics (see supporting Sec. I.B in File S1). This confirms that the force field approximation provides us with an accurate C_α_–C_α_ *versus* 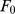 correlation curve (cf. triangles *versus* green dots).

Next, the validated nonlinear ruler can be used to analyze the experimental data after computing the average C_α_–C_α_ distances separately for the same sets of TDi, DO and different Ig protein disulfides that underly Fig. 3, see the horizontal dotted lines in Fig. 4. In the case of disulfide bonds in TDi, DO and interchain Ig proteins, their average C_α_–C_α_ distances turn out to be very similar, being about 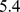, 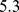 Å and 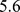 Å, respectively. In stark contrast, the mean C_α_–C_α_ distance of the intrachain disulfides from the Ig family turns out to be significantly longer, about 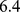 Å, and is no more far from the value of the significantly strained experimental macrocycle, [27], which is about 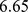 Å. It is thus concluded that the systematic strain of intrachain disulfide bridges in Ig proteins corresponds to tensile forces on the order of 0.1 nN (the conformational density landscapes for DEDS and cystine at 0.1 nN can be seen in Fig. S5 in file S1), whereas the TDi, DO and interchain Ig protein classes are characterized by unstrained disulfide bonds. It is worth mentioning that interchain disulfides have a few members overlapping with closed/closed conformations. Yet, they are not significantly strained since the average of their C_α_–C_α_ distance is 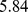 Å. A few of the intrachain disulfides, in turn, spread themselves in the region of open conformations. The average of their C_α_–C_α_ distance is 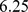 Å, which means that they are less strained than most of the intrachain disulfides (which adopt closed conformations), but, at the same time, more strained than TDi, DO and interchain disulfides.

## Discussion

On the basis of a simple method devised to quantitatively parameterize the internal strain in disulfide bridges as a result of tensile stress, it has been shown that disulfides belonging to native TDi, DO and interchain Ig protein disulfides are unstrained. As a consequence, they feature a high conformational diversity, and in particular avoid closed/closed conformations of the C–C–S–S dihedrals, i.e. 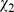/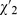 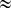 180°/180° (cf. Fig. S6 in file S1 at 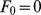 nN and Fig. S8 in file S1 for 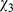), which are generally known to sterically hinder 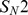
–type attack and thus redox reactions at the local level of the elementary chemical reaction. It thus follows that functional disulfides residing in DO, TDi proteins and functional interchain disulfides in Ig proteins are “conformationally active” towards redox reactions since their open conformations allow for easy access of the nucleophile and thus high reactivity.

The picture is vastly different for intrachain Ig protein disulfides, which overwhelmingly prefer closed/closed conformations of interstrand disulfide bridges instead. It should be noted in this context that those intrachain Ig protein disulfides which extend themselves into the open conformations also exhibit a longer C_α_–C_α_ distance, making this a characteristic feature. Here, this preference is directly correlated with internal strain, which manifests itself by a significant lengthening of the average distance between the two 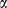
–carbons of the disulfide moiety when compared to that in both TDi and DO proteins. The tensile stress that distorts the intrastrand disulfide bridges in native intrachain Ig protein disulfides is shown to be on the order of 100 pN and is thus well within the range that is easily accessible to biomolecular processes [41]–[46].

The question that arises at this point is whether this mechanical activation could facilitate the reduction of an intrachain disulfide bond, thus neutralizing (or at least partially counteracting) the important local steric hindrance effects that a closed/closed conformation entails for the approach of a nucleophile close to the redox center. Within a simple mechanochemical picture, Bell's model [55], the increase of the rate of a chemical reaction due to applying an external force is found to be exponential in its magnitude 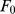 and can be roughly estimated from 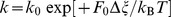, where 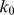 is the (usual thermal) rate at zero force and 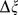 is a length parameter as comprehensively discussed [21]. Thus, a severalfold increase of the redox reaction rate is to be expected when applying forces on the order of 0.1 nN, as indeed measured by force–clamp AFM for disulfide bond reduction in proteins [25]. Given this phenomenon, the strained disulfide bonds in intrachain Ig protein disulfides can be called “mechanochemically active” since the exponentially accelerating mechanical work term 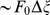 over–compensates local steric hindrance effects on nucleophilic attack of closed/closed with respect to open conformations. That said, we recall the well-established fact that intrachain disulfides in Ig domains are buried in the fold of the protein and that it is commonly accepted that they are non-reactive [56] because of the lack of solvent accessibility. Yet, some cases of reduction of this class of disulfides have already been documented [57], [58]. Besides, it has also been proposed that internal thiols in proteins can promote disulfide bond reductions in systems that exhibit functional “structural isoforms” [36]–[38]. It is thus concluded that mechanical activation is another important factor in promoting the redox reaction in those and other cases.

All things considered, the findings extracted from our constant-force simulations in conjunction with statistical analyses of available experimental data allow for a better understanding of the conformational diversity exhibited by disulfides in the first place. Moreover, they provide fresh insights into the interplay between conformation and reactivity of these moieties at the level of the elementary 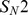 redox reaction. This will contribute to ongoing efforts devoted to achieve a detailed understanding of functional disulfide bonds at the molecular level.

## Methods

All force field MD simulations were carried out using GROMACS together with the OPLS-AA/SPC force fields. The metadynamics simulations were performed using PLUMED and GROMACS. The QM/MM MD simulations [54] of DEDS and the full ab initio MD simulations [54] of the macrocycle were done using the CPMD package. The force–clamp conditions were realized using the isotensional method [21]. A detailed account of the computational details including the references is provided in File S1.

## Supporting Information

File S1
**Combined Supporting Information file.** Figure S1. Macrocycle with the photoswitch, stiff stilbene, in its trans conformation leading to a strained disulfide bond. Figure S2. (a) Probability distribution function 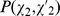 obtained from analyzing the X–ray crystal structures of disulfides in the Protein Data Bank (101087 observations) shown by contour lines with superimposed scatter plot of the conformational states of disulfide bonds in TDi proteins (40 observations) in blue circles, DO proteins (27 observations) as green diamonds and interchain Ig proteins (69 observations) as red squares. (b) Same as panel a but superimposing the scatter plot (circles) of the conformational states of disulfide bonds in intrachain Ig proteins (927 observations) showing the preference of closed/closed conformations, i.e. 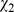/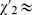 180°/180°. (c) Configuration of a representative interstrand disulfide bond (CYS A 132 – CYS A 192) in intact IgG1 monoclonal antibody (PDB ID 1IGY), which shows a closed/closed conformation that is typical to the preferred ones in panel b. The bridge is strained as a result of being suspended between the two strands shown in tube representation resulting into 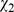/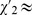 165°/178°. Figure S3. Probability distribution functions 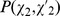 obtained from metadynamics simulations for DEDS at zero force and 0.3 nN in panels a and b, respectively. Figure S4. Probability distribution functions 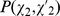 obtained from metadynamics simulations for cystine at zero force and 0.3 nN in panels a and b, respectively. Figure S5. Probability distribution functions 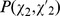 obtained from metadynamics simulations for DEDS and cystine at 0.1 nN force in panels a and b, respectively. Figure S6. Total open character according to the 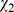 and 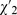 dihedral angles (see text) for the model polypeptide, cystine and DEDS as a function of constant external force. The inset shows the closed–closed (the right picture, 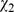 and 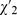 in the range 180
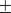
50°) and the open–open conformation (the left picture, 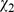 and 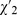 all other values for the dihedral apart from 180
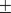
50°) in these dihedrals. These data have been extracted from 150, 300, 1300 ns of zero force and force–clamp MD simulations performed on DEDS, cystine, and the model polypeptide, respectively. Figure S7. Mechanical coordinate, 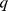, for the model polypeptide, cystine and DEDS as a function of constant external force. Figure S8. Central disulfide dihedral angle C–S–S–C, 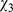, for the model polypeptide, cystine and DEDS as a function of constant external force; the inset visualizes the respective torsional degree of freedom. Note that this behavior, which is very similar for all three systems, is similar to the one found earlier for a cystine molecule.(PDF)
