# Supplementary material for: The Effect of Tensile Stress on the Conformational Free Energy Landscape of Disulfide Bonds
Source: PLoS One. 2014 Oct 6;9(10):e108812. doi: 10.1371/journal.pone.0108812 (PMC4186883; doi:10.1371/journal.pone.0108812)
Supplement: File S1 — Combined Supporting Information file. Figure S1. Macrocycle with the photoswitch, stiff stilbene, in its trans conformation leading to a strained disulfide bond. Figure S2. (a) Probability distribution function obtained from analyzing the X–ray crystal structures of disulfides in the Protein Data Bank (101087 observations) shown by contour lines with superimposed scatter plot of the conformational states of disulfide bonds in TDi proteins (40 observations) in blue circles, DO proteins (27 observations) as green diamonds and interchain Ig proteins (69 observations) as red squares. (b) Same as panel a but superimposing the scatter plot (circles) of the conformational states of disulfide bonds in intrachain Ig proteins (927 observations) showing the preference of closed/closed conformations, i.e. / 180°/180°. (c) Configuration of a representative interstrand disulfide bond (CYS A 132 – CYS A 192) in intact IgG1 monoclonal antibody (PDB ID 1IGY), which shows a closed/closed conformation that is typical to the preferred ones in panel b. The bridge is strained as a result of being suspended between the two strands shown in tube representation resulting into / 165°/178°. Figure S3. Probability distribution functions obtained from metadynamics simulations for DEDS at zero force and 0.3 nN in panels a and b, respectively. Figure S4. Probability distribution functions obtained from metadynamics simulations for cystine at zero force and 0.3 nN in panels a and b, respectively. Figure S5. Probability distribution functions obtained from metadynamics simulations for DEDS and cystine at 0.1 nN force in panels a and b, respectively. Figure S6. Total open character according to the and dihedral angles (see text) for the model polypeptide, cystine and DEDS as a function of constant external force. The inset shows the closed–closed (the right picture, and in the range 18050°) and the open–open conformation (the left picture, and all other values for the dihedral apart from 18050°) in [file pone.0108812.s001.pdf]

Supplementary Information: The effect of tensile stress on the conformational free energy landscape of disulfide bonds

Padmesh Anjukandi[(1)],* Przemyslaw Dopieralski[(1,2)],
Jordi Ribas–Arino[(1,3)], and Dominik Marx[(1)]

[(1)] *Lehrstuhl für Theoretische Chemie,*
Ruhr–Universität Bochum, 44780 Bochum, Germany

[(2)] *Permanent Address: Faculty of Chemistry,*
University of Wroclaw, Joliot–Curie 14, 50-383 Wroclaw, Poland

[(3)] *Current Address: Departament de Química Física and IQTCUB,*
Universitat de Barcelona, Av. Diagonal 645, 08028, Barcelona, Spain

I. SIMULATION METHODS AND COMPUTATIONAL DETAILS

A. Equilibrium and Metadynamics Force Field Simulations

All force field molecular dynamics (MD) simulations were carried out with the software suite `GROMACS`–4.5.3.[1,2] The OPLS–AA[3,4] all–atom force field was applied for simulating the polypeptide as well as cystine and diethyl disulfide (DEDS) together with using the SPC water model[5] for explicit solvation. The simulations were run in the NVT ensemble with periodic boundary conditions. The temperature of the systems was kept constant at 300 K by coupling to Nosé–Hoover chain thermostats[6], an integration time step of 2 fs was used, trajectory coordinates were recorded every 200 fs for analysis, and all bond lengths were constrained using LINCS.[7] The electrostatic interactions were incorporated using a reaction field method[8] that has been shown to perform well for the thermodynamics of protein folding[9] and thus for computing conformational probability density maps are required here.

In order to converge the sampling of the conformational χ_2/χ_2'–space, metadynamics acceleration[10,11] was carried out using the `PLUMED` plugin[12] together with `GROMACS`–4.5.5. During metadynamics, the conformational sampling of the χ_2 and χ_2' torsional angles, being the collective variables for metadynamics, as defined in Fig. 1 of the main text were accelerated for all three systems using Gaussian biasing functions (height: 0.23 kcal/mol, width: 0.1 radians) and deposited every 10 ps for a total simulation length of 300 ns. We assume that by sampling up to a free energy of $\approx$ 17 kcal/mol via metadynamics almost all possible conformations of the disulfides should be properly sampled as the highest strain energy in a disulfide moiety is found to be of that order or magnitude[13,14] (corresponding to the Cys37–Cys54 bond of 3–oxoacyl–acyl carrier protein reductase from *Thermotoga maritima*, PDB ID 1O5I). In the case of DEDS and cystine, we assume this value to be even lower as they are free at their termini and hence not that much constrained as disulfide bonds in proteins.

DEDS, cystine and the polypeptide model Ile–Cys–Leu–Ser–Glu–Pro–Asp–Val–His–Cys–Gln (with the disulfide bond between the 2nd and 10th cysteine residue, were generated using PyMOL[15], minimized (1000 steps using the steepest descent algorithm) and subsequently equilibrated. The species where solvated with SPC water in a periodic box of (3.0 x 3.0 x 3.0) nm^3 for DEDS and cystine whereas a simulation box of (5.0 x 4.0 x 4.0) nm^3 was used

for the polypeptide using 886, 880, and 2633 solvent molecules, respectively In addition to the solvent, 10 Na^+ and 9 Cl^- ions were also added to the protein model system in order to keep the system neutral. The termini of the polypeptide were capped with hydrogens as to avoid any Coulomb attraction bias on the conformational free energies when using such a rather short peptide.

Force–clamp MD simulations of these species (that have been equilibrated at zero force) were then performed using the same computational approach as described above by explicitly including constant forces[16] thus sampling isotensional force–transformed free energy surfaces[17,18]; see Ref.[19] for a detailed review on these computational techniques. In the present case, constant stretching forces from zero up to $F_0 = 2.0$ nN where applied to the terminal N and C atoms (of cystine and the polypeptide) and the two methyl C atoms (for DEDS) along the space–fixed x axis, applied in a systematically increasing manner for 2 ns in opposite directions along the x-coordinate to equilibrate the stretched molecule. Further, production runs were carried out for DEDS, Cystine and Polypeptide for 150, 300 and 1300 ns respectively and assesed all the necessary datas. `VMD`[20] was used to analyze the dihedrals, the mechanical coordinate q (i.e. the distance between the atoms on which the external force acts), and various other structural parameters. The probability distribution functions and free energy surfaces were obtained from the isotensional metadynamics simulations and were visualized using the commercial software package `MATLAB`–7.6.0 (The MathWorks Inc., Natick, MA, 2000). The populations of the open conformers in all these models were computed. The "openness" and "closed" nature of the χ torsionals of DEDS were benchmarked by the specific values of their internal dihedrals.[18] In the case of χ_3 angle, any conformation within the dihedral range of 180±50° defines the *trans* conformation and all others defines a *normal* standard conformation of χ_3. Similarly, χ_2 and χ_2' conformations with dihedral angle in the range 180±50° refers to a *closed* conformation and all other values for these dihedrals refers to an *open* conformation. The total closed nature in χ_2 and χ_2' angles were then obtained and the openness was determined by taking the difference to unity.

B. QM/MM Reference Simulations of DEDS

In order to validate the performance of the intramolecular force field when stretching molecules we carried out reference calculations by treating the smallest system, DEDS, us-

ing electronic structure based ("on the fly") MD, while sticking to a force field representation of the chemically inert explicit solvent. Thus, hybrid QM/MM MD simulations have been carried out upon applying a constant external force F_0 as before using the well–established `CPMD/GROMOS` coupling scheme.[21–23]. These dynamical calculations are based on ab initio molecular dynamics (AIMD)[23] using the efficient Car–Parrinello[24] propagation scheme as implemented in the `CPMD` program package[25]. For the QM system, the BLYP density functional[26,27] and a plane wave basis set with a cutoff of 70 Ry together with normconserving pseudopotentials[28] was utilized in a nonperiodic cubic box of 14 Å. The MM system consisted of 70 water molecules and an OH^- ion represented by the SPC force field.[5] These simulations were performed in the canonical ensemble at 300 K using Nosé–Hoover chain thermostats[6] in order to control the kinetic energy of the nuclei as well as the fictitious kinetic energy of the orbitals. An AIMD time step of 6 a.u.($\approx$ 0.14 fs) was used for the integration of the Car–Parrinello equations of motion using a fictitious mass parameter for the orbitals of $\mu = 700$ a.u. together with the atomic masses of all hydrogens substituted by deuterium masses to allow for larger fictitious orbital mass and thus a larger time step resulting into more efficient AIMD propagation.[23] For each force value, the system was equilibrated initially for 2 ps followed by production runs of 200 ps for analyses.

C. QM Simulations on Disulfide Macrocycle

In previous experiments[29], chemically engineered macrocycles, so–called "molecular force probes",[30,31] have been used in order to generate internal mechanical stress that strains the disulfide bond that is embedded in the macrocycle.[19] For such systems the tensile force corresponding to the produced strain has been extracted by a validated approach.[29,31]

In order to compute the corresponding C_α–C_α distance at room temperature, an AIMD[23] simulation of an isolated disulfide macrocycle (macrocycle 9[29], has been performed. Importantly, no external force must be applied to the macrocyle, where strain is generated purely intramolecularly due to the photoswitch, stiff stilbene, being in the cis/trans conformation, see Fig. 1. These AIMD simulations have been conducted as explained in the previous section using a 20 Å cubic box, proper hydrogen masses, and thus a timestep of 3 a.u. with $\mu = 400$ a.u. was set to be a cubic box of 20 Å. Again, the system was initially equilibrated for 2 ps followed by 120 ps for computing the average C_α–C_α distance.

II. SUPPLEMENTARY FIGURES

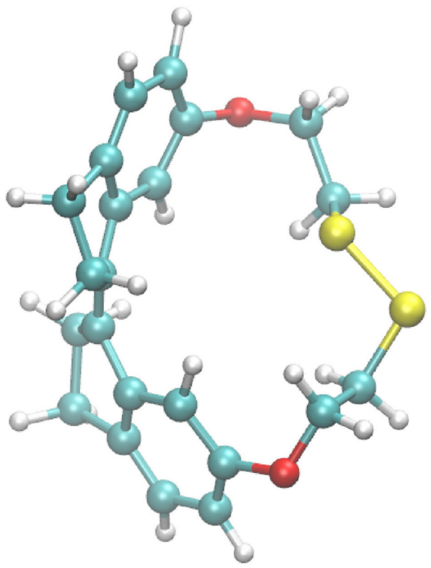

FIG. 1: Macrocycle[29] with the photoswitch, stiff stilbene, in its trans conformation leading to a strained disulfide bond.

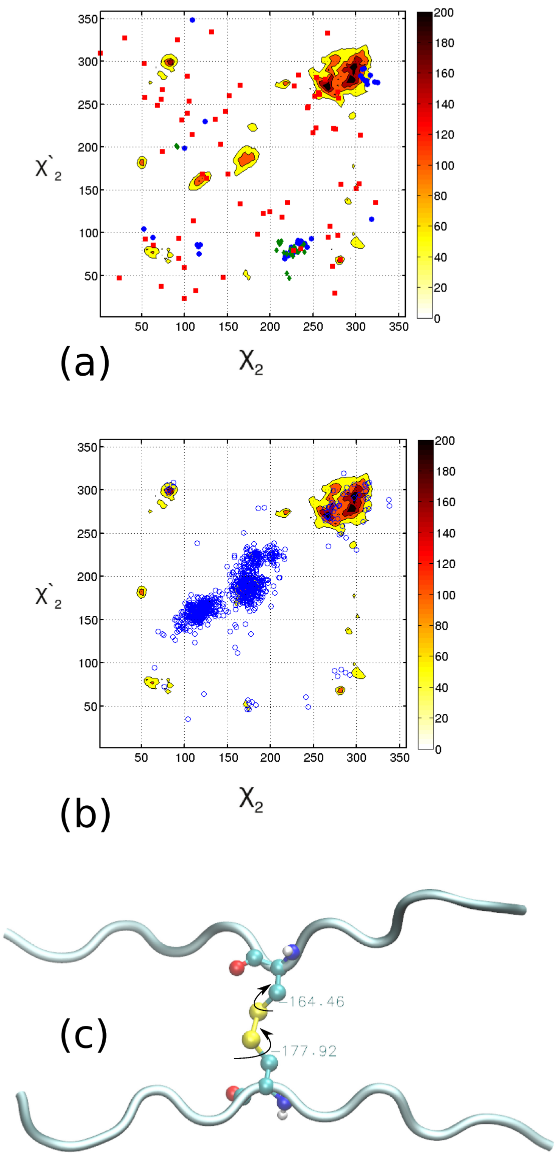

FIG. 2: (a) Probability distribution function $P(\chi_2, \chi_2')$ obtained from analyzing the X–ray crystal structures of disulfides in the Protein Data Bank (101087 observations) shown by contour lines with superimposed scatter plot of the conformational states of disulfide bonds in TDi proteins (40 observations) in blue circles, DO proteins (27 observations) as green diamonds and interchain Ig proteins (69 observations) as red squares. (b) Same as panel a but superimposing the scatter plot (circles) of the conformational states of disulfide bonds in intrachain Ig proteins (927 observations) showing the preference of closed/closed conformations, i.e. $\chi_2/\chi_2' \approx 180°/180°$. (c) Configuration of a representative interstrand disulfide bond (CYS A 132 – CYS A 192) in intact IgG1 monoclonal antibody (PDB ID 1IGY), which shows a closed/closed conformation that is typical to the preferred ones in panel b. The bridge is strained as a result of being suspended between the two strands shown in tube representation resulting into $\chi_2/\chi_2' \approx 165°/178°$.

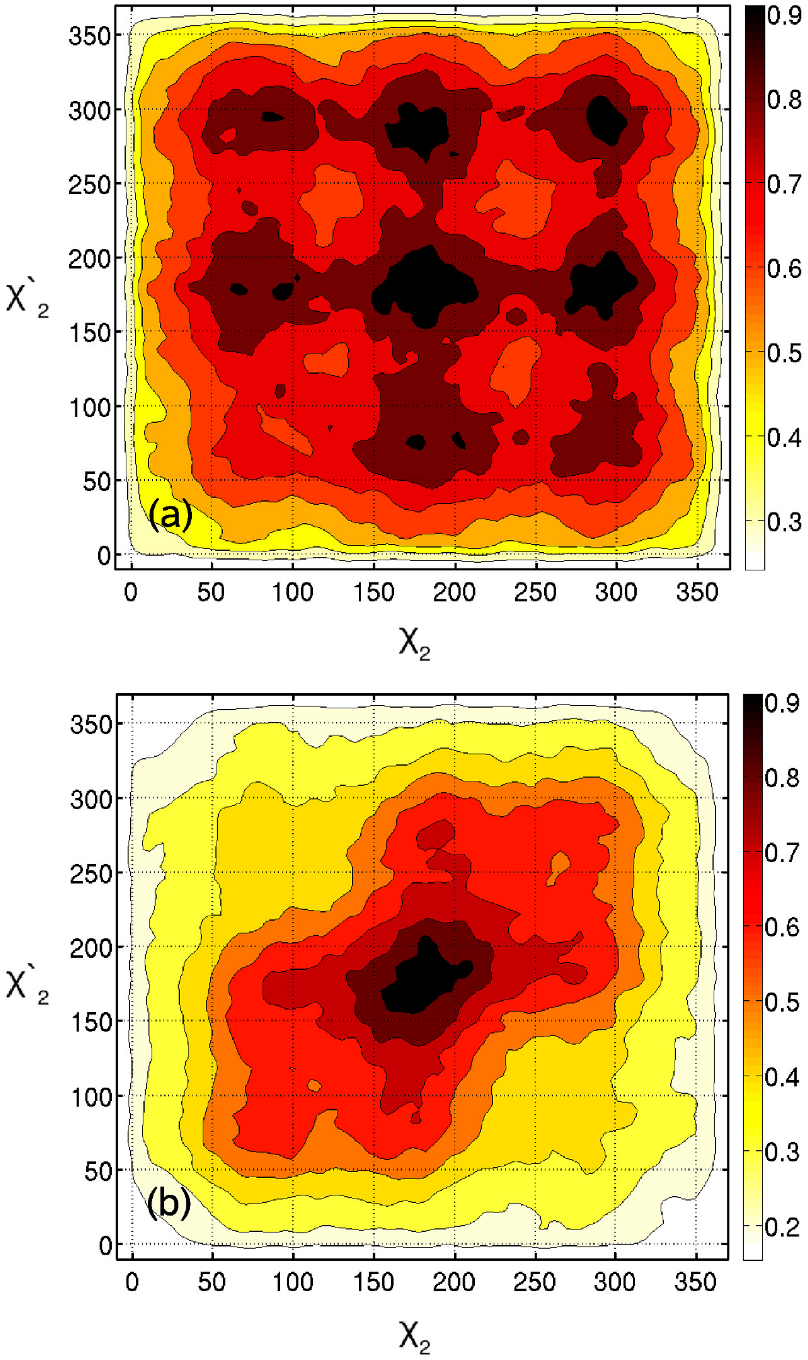

FIG. 3: Probability distribution functions $P(\chi_2, \chi_2')$ obtained from metadynamics simulations for DEDS at zero force and 0.3 nN in panels a and b, respectively.

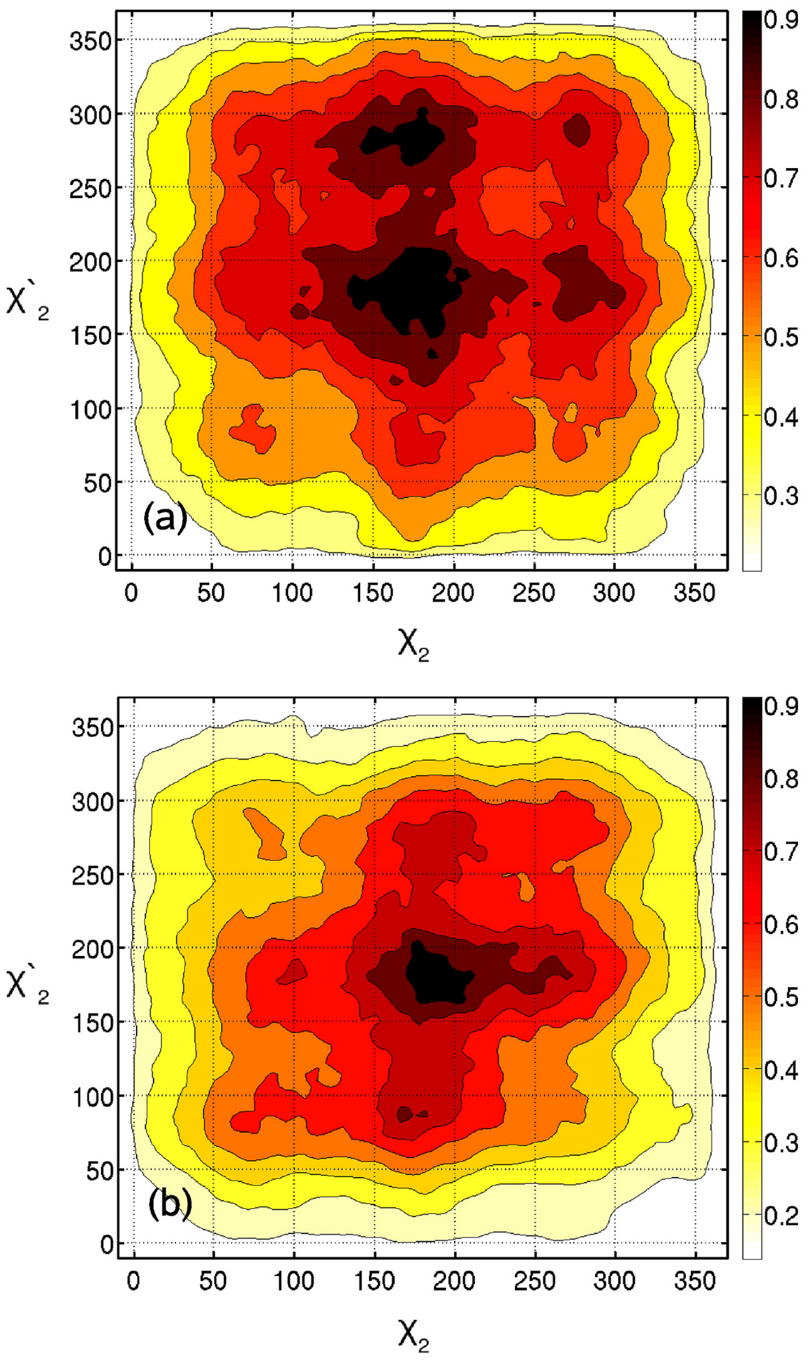

FIG. 4: Probability distribution functions $P(\chi_2, \chi_2')$ obtained from metadynamics simulations for cystine at zero force and 0.3 nN in panels a and b, respectively.

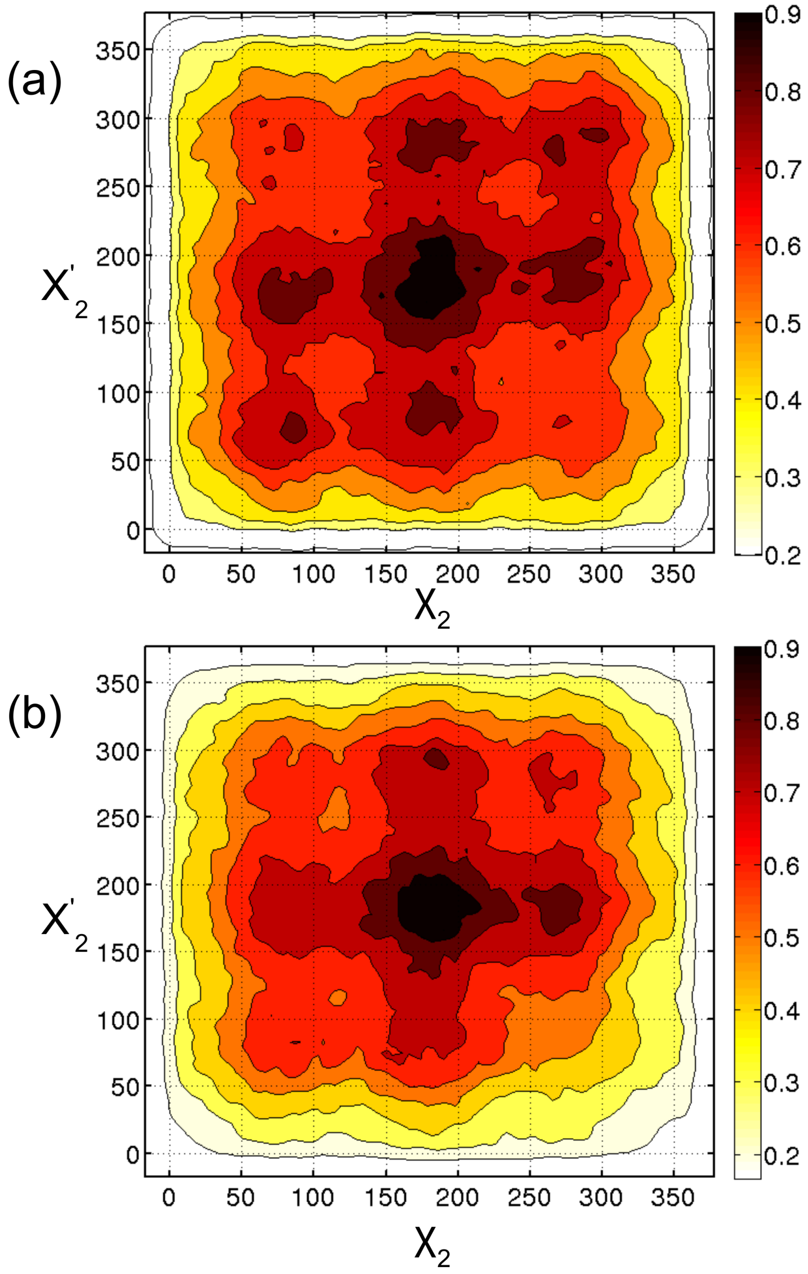

FIG. 5: Probability distribution functions $P(\chi_2, \chi_2')$ obtained from metadynamics simulations for DEDS and cystine at 0.1 nN force in panels a and b, respectively.

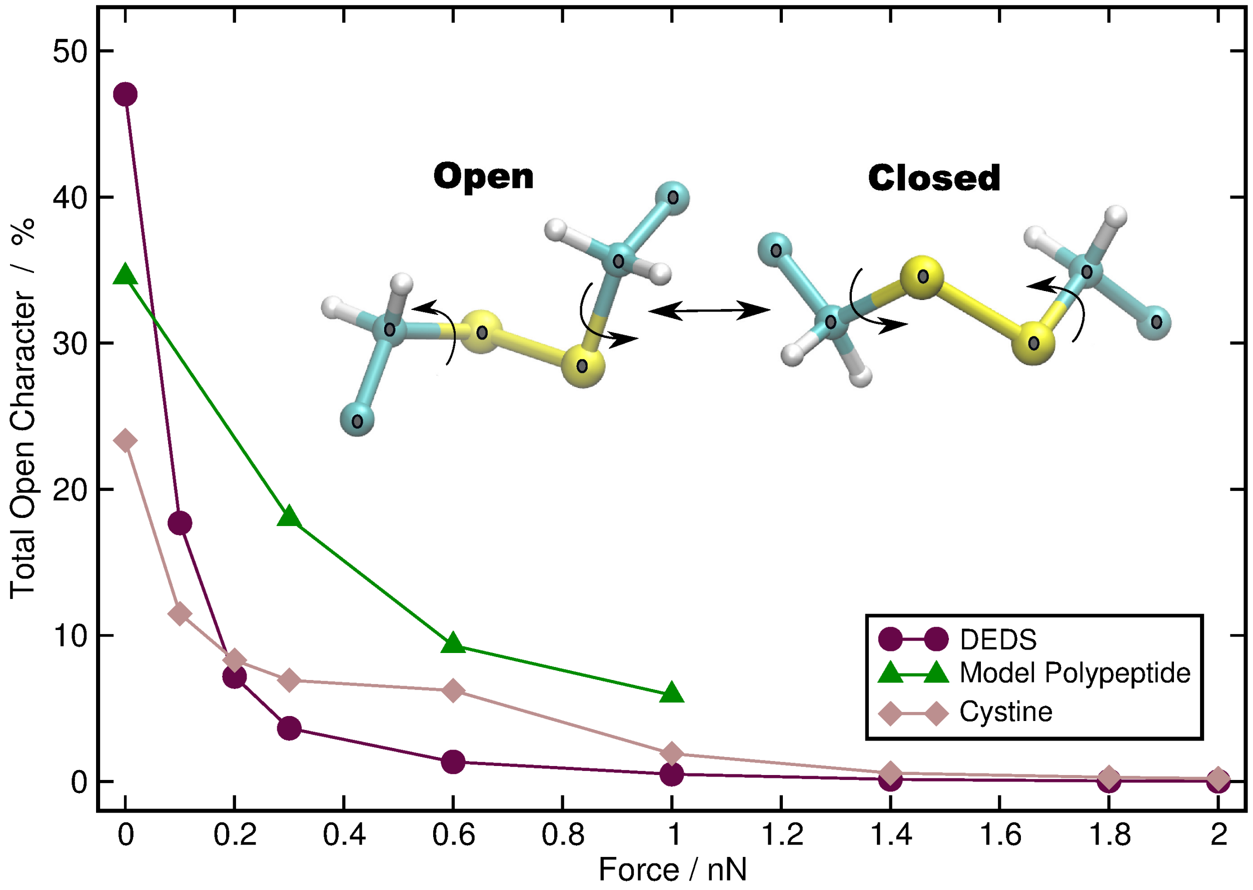

FIG. 6: Total open character according to the χ_2 and χ_2' dihedral angles (see text) for the model polypeptide, cystine and DEDS as a function of constant external force. The inset shows the closed–closed (the right picture, χ_2 and χ_2' in the range 180$\pm$50°) and the open–open conformation (the left picture, χ_2 and χ_2' all other values for the dihedral apart from 180$\pm$50°) in these dihedrals. These data have been extracted from 150, 300, 1300 ns of zero force and force–clamp MD simulations performed on DEDS, cystine, and the model polypeptide, respectively.

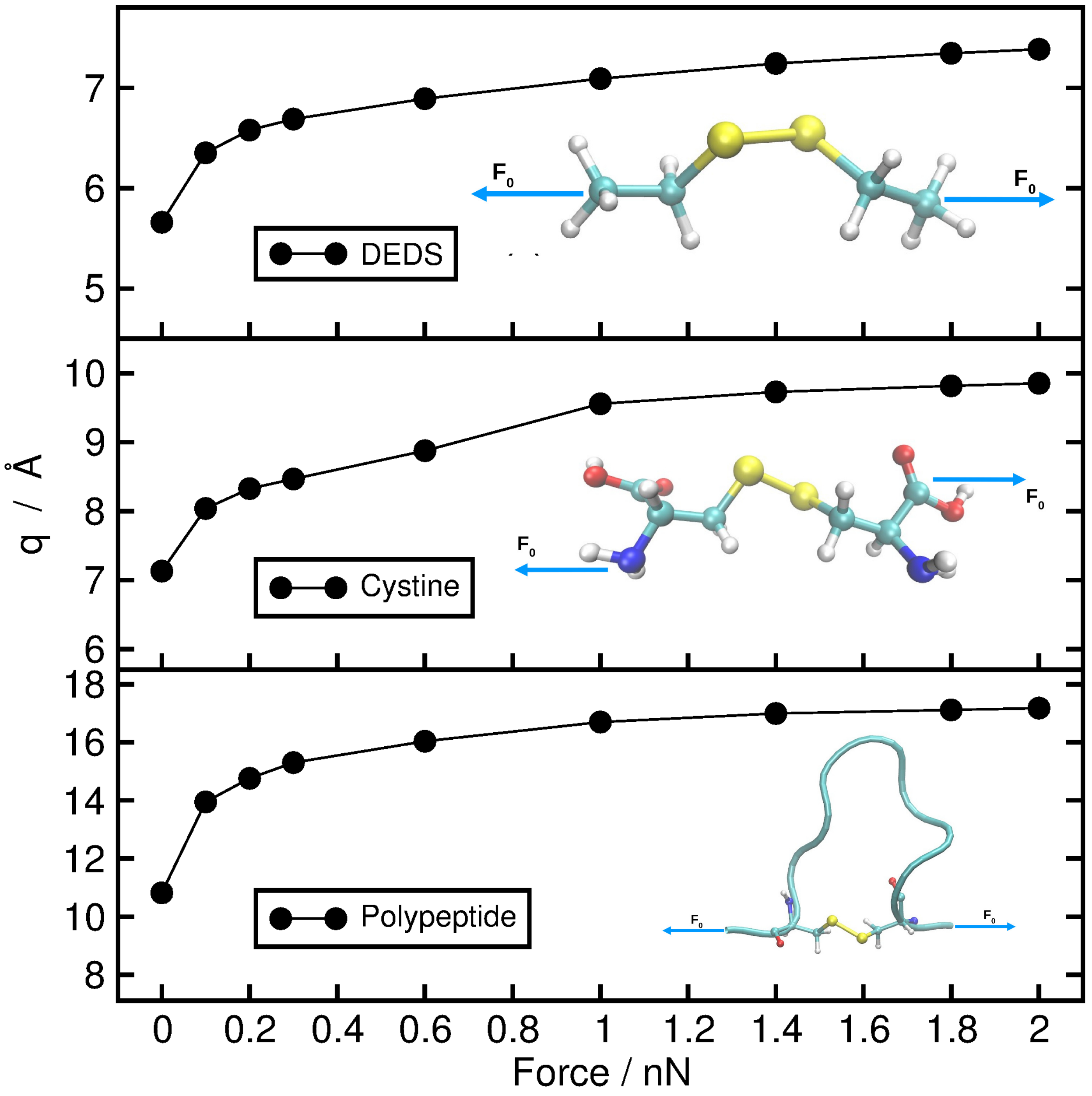

FIG. 7: Mechanical coordinate, q, for the model polypeptide, cystine and DEDS as a function of constant external force.

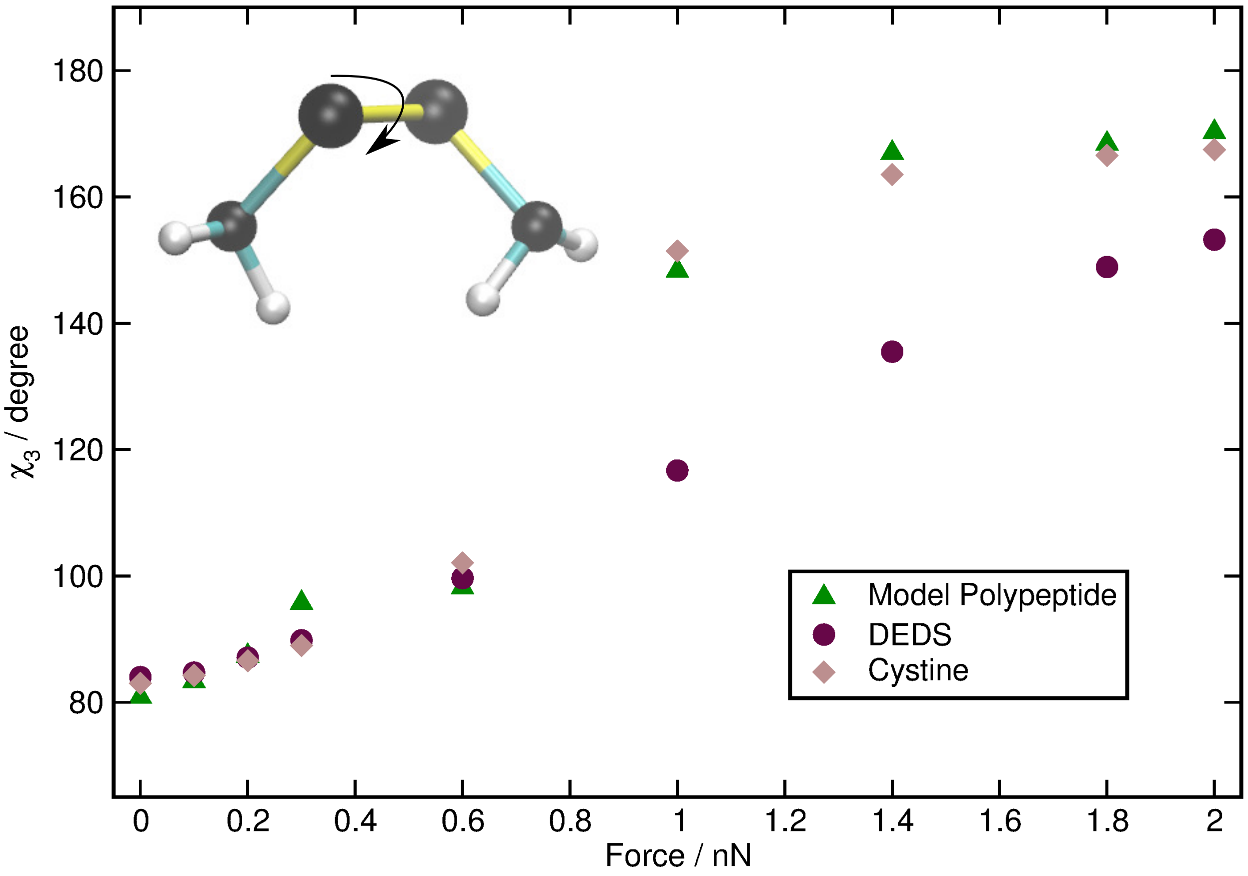

FIG. 8: Central disulfide dihedral angle C–S–S–C, χ_3, for the model polypeptide, cystine and DEDS as a function of constant external force; the inset visualizes the respective torsional degree of freedom. Note that this behavior, which is very similar for all three systems, is similar to the one found earlier for a cystine molecule.[32]

* Electronic address: padmesh.anjukandi@theochem.rub.de

[1] Berendsen, H. J. C., Spoel, D. V. D. & Drunen, R. V. Gromacs: A message–passing parallel molecular dynamics implementation. *Comp. Phys. Commun.* **91**, 43–56 (1995).

[2] Hess, B., Kutzner, C., van der Spoel, D. & Lindahl, E. GROMACS 4: Algorithms For Highly Efficient, Load–Balanced, and Scalable Molecular Simulation. *J. Chem. Theory and Comput.* **4**, 435–447 (2008).

[3] Jorgensen, W. L. & Tirado-Rives, J. The opls potential functions for prpteins – energy minimizations for crystals of cyclic–peptides and crambin. *J. Am. Chem. Soc.* **110**, 1657–1666 (1988).

[4] l. Jorgensen, W., Maxwell, D. S. & Tirado-Rives, J. Development and Testing of the OPLS All–Atom Force Field on Conformational Energetics and Properties of Organic Liquids. *J. Am. Chem. Soc.* **118**, 11225–11236 (1996).

[5] Berendsen, H. J. C., andW. F. van Gunsteren, J. P. M. P. & Hermans, J. Interaction models for water in relation to protein hydration. In Pullman, B. (ed.) *Intermolecular Forces*, vol. 1, 331–342 (D. Reidel Publishing Company, Dordrecht, 1981).

[6] Martyna, G. J., Klein, M. L. & Tuckerman, M. Nosé-Hoover chains: the canonical ensemble via continuous dynamics. *J. Chem. Phys.* **97**, 2635–2643 (1992).

[7] Hess, B., Bekker, H., Berendsen, H. J. C. & Fraajie., J. G. E. M. Lincs: A linear constraint solver for molecular simulations. *BMC Structural Biology* **18**, 1463–1472 (1997).

[8] Tironi, I. G., Sperb, R., Smith, P. E. & van Gunsteren, W. F. A generalized reaction feld method for molecular dynamics simulations. *J. Phys. Chem. B* **109**, 21322–21328 (2005).

[9] Baumketner, A. & Shea, J. E. Influence of different treatments of electrostatic interactions on the thermodynamics of folding of peptides. *J. Phys. Chem. B* **109**, 21322–21328 (2005).

[10] Laio, A. & Parrinello, M. Escaping free-energy minima. *Proc. Natl. Acad. Sci. USA* **99**, 12562–12566 (2002).

[11] Iannuzzi, M., Laio, A. & Parrinello, M. Efficient exploration of reactive potential energy surfaces using Car-Parrinello Molecular Dynamics. *Phys. Rev. Lett.* **90**, 238302–1–4 (2003).

[12] Bonomi, M. *et al.* Plumed: A portable plugin for free-energy calculations with molecular dynamics. *Comp. Phys. Commun.* **180**, 1961–1972 (2009).

[13] Schmidt, B., Ho, L. & Hogg, P. J. Allosteric disulfide bonds. *Biochemistry* **45**, 7429–7433 (2006).

[14] Schmidt, B. & Hogg, P. J. Search for allosteric disulfide bonds in nmr structures. *BMC Structural Biology* **7**, 49 (2007).

[15] The pymol molecular graphics system. *Schrödinger. LLC* **Version 1.2r3pre**.

[16] Ribas-Arino, J., Shiga, M. & Marx, D. Understanding covalent mechanochemistry. *Angew. Chem. Int. Ed.* **48**, 4190–4193 (2009).

[17] Dopieralski, P., Ribas-Arino, J. & Marx, D. Force–transformed free energy surfaces and trajectory shooting simulations reveal the mechano–stereochemistry of cyclopropane ring–opening reactions. *Angew. Chem. Int. Ed.* **50**, 7105–7108 (2011).

[18] Dopieralski, P. *et al.* The janus–faced role of external forces in mechanochemical disulfide bond cleavage. *Nat. Chem.* **5**, 685–691 (2013).

[19] Ribas-Arino, J. & Marx, D. Covalent mechanochemistry: theoretical concepts and computational tools with applications to molecular nanomechanics. *Chem. Rev.* **112**, 5412–5487 (2012).

[20] Humphrey, W., Dalke, A. & Schulten, K. Vmd: Visual molecular dynamics. *J. Mol. Graphics* **14**, 33–38 (1996).

[21] Laio, A., VandeVonde, J. & Rothlisberger, U. A Hamiltonian electrostatic coupling scheme for hybrid Car-Parrinello molecular dynamics simulations. *J. Chem. Phys.* **116**, 6941–6947 (2002).

[22] Laio, A., VandeVonde, J. & Rothlisberger, U. D-RESP: dynamically generated electrostatic potential derived charges from quantum mechanics/molecular mechanics simulations. *J. Phys. Chem. B* **106**, 7300–7307 (2002).

[23] Marx, D. & Hutter, J. *Ab initio molecular dynamics: basic theory and advanced methods* (Cambridge University Press, Cambridge, 2009).

[24] Car, R. & Parrinello, M. Unified Approach for Molecular Dynamics and Density-Functional Theory. *Phys. Rev. Lett.* **55**, 2471–2474 (1985).

[25] J. Hutter *et al.* `CPMD` *Program Package, see* `http://www.cpmd.org`. see `http://www.cpmd.org`.

[26] Becke, A. D. Density-functional exchange-energy approximation with correct asymptotic behavior. *Phys. Rev. A* **38**, 3098–3100 (1988).

[27] Lee, C., Yang, W. & Parr, R. G. Development of the Colle-Salvetti correlation-energy formula into a functional of the electron density. *Phys. Rev. B* **37**, 785–789 (1988).

[28] Troullier, N. & Martins, J. L. Efficient pseudopotentials for plane-wave calculations. *Phys. Rev. B* **43**, 1993–2006 (1991).

[29] Kucharski, T. J. *et al.* Kinetics of thiol/disulfide exchange correlate weakly with the restoring force in the disulfide moiety. *Angew. Chem. Int. Ed.* **48**, 7040–7043 (2009).

[30] Yang, Q. Z. *et al.* A molecular force probe. *Nat. Nanotech.* **4**, 302–306 (2009).

[31] Huang, Z. *et al.* Method to Derive Restoring Forces of Strained Molecules from Kinetic Measurements. *J. Am. Chem. Soc.* **131**, 1407–1409 (2009).

[32] Baldus, I. B. & Gräter, F. Mechanical force can fine-tune redox potentials of disulfide bonds. *Biophys. J.* **102**, 622–629 (2012).
